# Supplementary material for: Causal Relationship between Aging and Anorexia Nervosa: A White-Matter-Microstructure-Mediated Mendelian Randomization Analysis
Source: Biomedicines. 2024 Aug 16;12(8):1874. doi: 10.3390/biomedicines12081874 (PMC11351342; doi:10.3390/biomedicines12081874)
Supplement: Supplementary file 1 [file biomedicines-12-01874-s001.zip › Supplementary Figure/Figure S8/Leaveoneout plot of MR analysis for TL to ubm-b-1620.pdf]

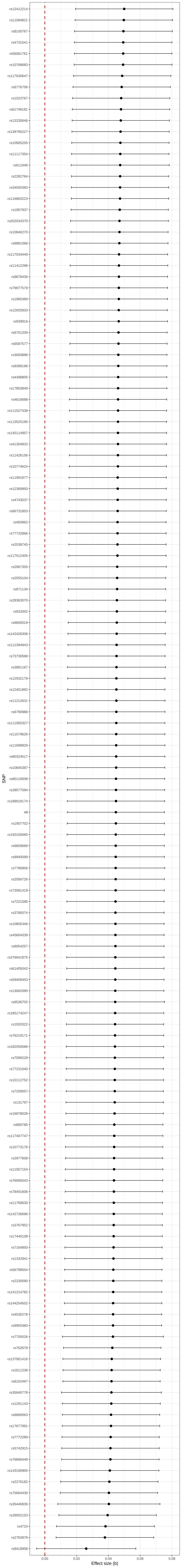
SNP
rs12412214
rs11584821
rs8105767
rs4731541
rs56061761
rs10768683
rs117630647
rs6776756
rs1023767
rs61748181
rs13230646
rs139795227
rs10905255
rs11117354
rs611646
rs2282764
rs34550383
rs116863223
rs1957937
rs202034370
rs10840270
rs6881568
rs117034449
rs11412296
rs9878436
rs79977579
rs1985369
rs12925933
rs939916
rs6751209
rs6587577
rs3093888
rs9398196
rs4498805
rs17803849
rs4616688
rs111527438
rs113525195
rs145114957
rs41304832
rs11426156
rs10774624
rs11991877
rs12369950
rs4743037
rs66731853
rs450962
rs77732866
rs2538745
rs117512405
rs2967355
rs2555104
rs871134
rs28363070
rs932002
rs9600019
rs142426306
rs112394943
rs73730598
rs3891167
rs12932179
rs12451892
rs11212631
rs6790988
rs111950327
rs11579626
rs11699829
rs80324517
rs10845387
rs80116508
rs28577594
rs188918174
All
rs1907702
rs150150565
rs6659669
rs9940099
rs7790856
rs2056726
rs73581419
rs7221585
rs3785074
rs10805346
rs45604339
rs6054257
rs376641875
rs61405042
rs59409453
rs13062095
rs6536702
rs185174247
rs1003322
rs76219171
rs182059586
rs7099229
rs77231040
rs10112752
rs7209057
rs131797
rs16978028
rs869785
rs117407747
rs10773176
rs2977608
rs11557154
rs76065543
rs78491606
rs11769630
rs142730696
rs3767952
rs17445108
rs7164950
rs1332941
rs56799554
rs2230590
rs141214782
rs144204502
rs4530278
rs9955360
rs7705526
rs762679
rs137901416
rs1611236
rs8102497
rs35640778
rs1291143
rs6669563
rs17677991
rs7772289
rs5742915
rs76666449
rs143190905
rs2276182
rs75664430
rs35446936
rs28502153
rs4724
rs2763979
rs9419958
0.00
0.02
0.04
0.06
0.08
Effect size (b)
